# Oat Cell Carcinoma Lung Presenting as Chest Wall Swelling

**DOI:** 10.7759/cureus.17499

**Published:** 2021-08-27

**Authors:** Manish Wadhwa, Joanna J Ekabua, Aisha O Adigun, Gaurav Singla

**Affiliations:** 1 Surgery, Maharishi Markandeshwar Medical College and Hospital, Solan, IND; 2 Infectious Disease, University of Louisville School of Medicine, Louisville, USA; 3 Radiology, Maharishi Markandeshwar Medical College and Hospital, Solan, IND

**Keywords:** small-cell lung carcinoma, oat cell carcinoma lung, chest wall swelling, cigarette smoking, distant metastasis, palliative care, surgical oncology

## Abstract

Chest wall swelling originating from lung is an uncommon phenomenon that makes its diagnosis a challenging task. We present a case where an 82-year-old man, a lifetime smoker, presented with a chest swelling. The origin of the swelling was an extension of a peripherally located lung malignancy diagnosed with the help of contrast-enhanced CT chest and confirmed as oat(or anaplastic) cell carcinoma on histology. After complete workup it was diagnosed as metastatic small cell carcinoma lung. Patient was managed with palliative chemoradiotherapy.

## Introduction

Chest wall swellings may not always arise from the constituents of chest wall but sometimes they result from the extension of contents of underlying viscera. Chest wall swelling with underlying lung involvement is a rare finding. Detailed history and careful physical examination along with a high index of suspicion is required to know the origin of such swellings. Chest wall invasion in patients already diagnosed with non-small cell lung carcinoma (NSCLC) is not an uncommon finding but small cell carcinoma lung (SCLC) presenting as chest wall swelling is a very rare phenomenon. In this article, we report a patient who presented in surgery outpatient clinic with chest wall swelling later on diagnosed to be a case of advanced small cell carcinoma lung (SCLC).

## Case presentation

**Figure 1:**
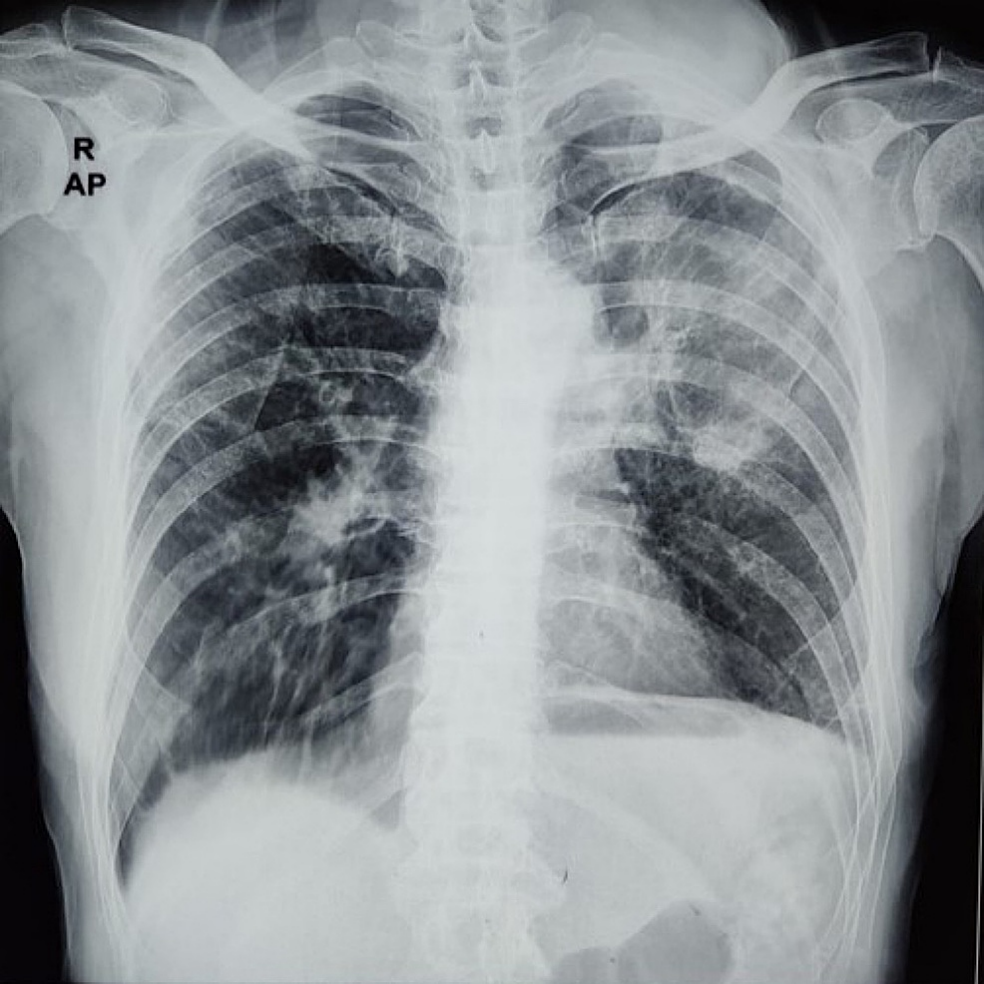
Chest X-ray postero-anterior view suggestive of a large opacity on the left side chest.

An 82-year-old male, farmer by occupation, presented with painful swelling in the left side of chest for last eight months. Swelling started spontaneously and progressively increased in size. During the last eight months, patient had an on and off mild cough, four to five episodes of hemoptysis and also complained about loosening of clothes. He had no other significant past medical and family history. He was a heavy smoker who smoked one to two packs of ‘bidi’ (a type of cheap cigarette containing unprocessed tobacco wrapped in a leaf) for 50 years. He was being treated symptomatically at local primary health care centre but his symptoms did not improve and reported to our hospital for further management after eight months of symptoms onset. On examination, 8 cm x 6 cm, non-pulsatile swelling present over the left 3rd to 6th intercostal space. Swelling was tender, hard in consistency, had well-defined margins, fixed to skin and underlying structures, had ulcerated surface along with necrotic patches. Ipsilateral supraclavicular lymph nodes were enlarged (approx 4 cm x 3 cm), hard in consistency, fixed and had a smooth surface with regular margins. There were no other swellings in any other part of the body. His review of systems was unremarkable. Routine hemogram was within normal limits. ESR was not elevated and Montoux test was negative. Sputum for acid-fast bacilli was negative on Zeihl-Neelsen staining. Chest X-ray postero-anterior view revealed large left lung mass with the probable invasion of chest wall and pleura without any bony involvement (Figure 1). An ill-defined lesion was also reported on the right side in X-ray chest PA view. However, no lesion was found on the right side chest on the CECT chest.

**Figure 2:**
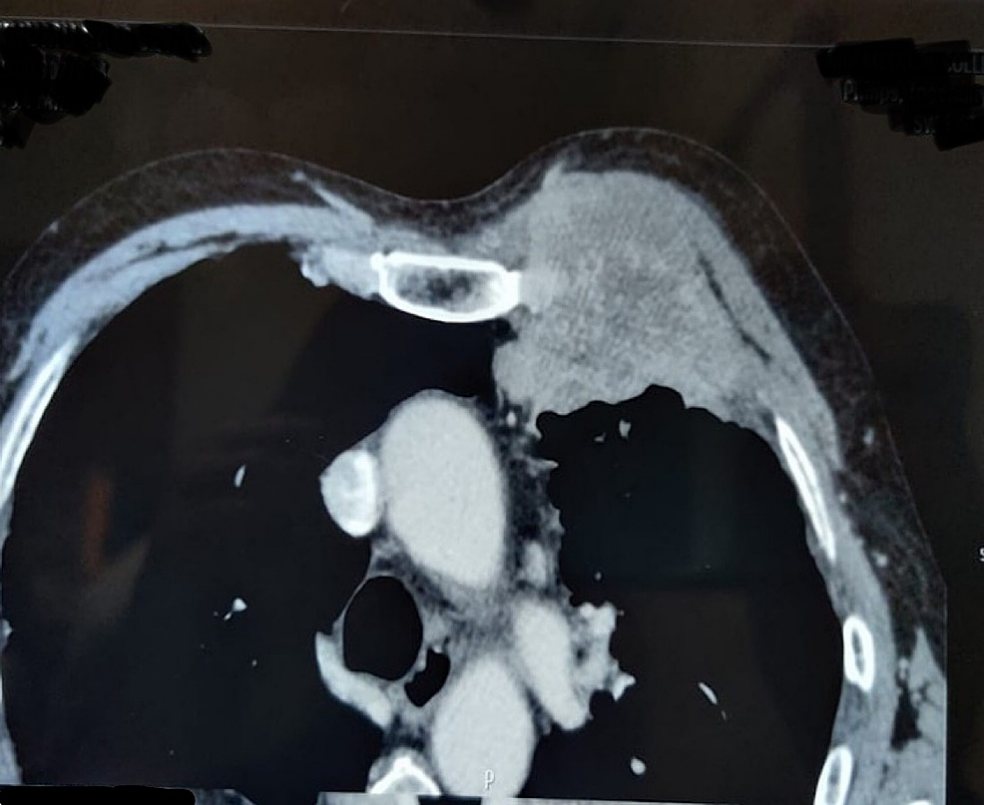
CECT chest showing chest wall infiltration by an ill-defined mass arising from left lung parenchyma. CECT: contrast-enhanced CT.

CECT Thorax detected ill-defined peripheral heterogenous mass anterior segment of the left upper lobe with chest wall invasion (without any bony involvement) likely s/o peripheral lung malignancy (Figure 2).

**Figure 3:**
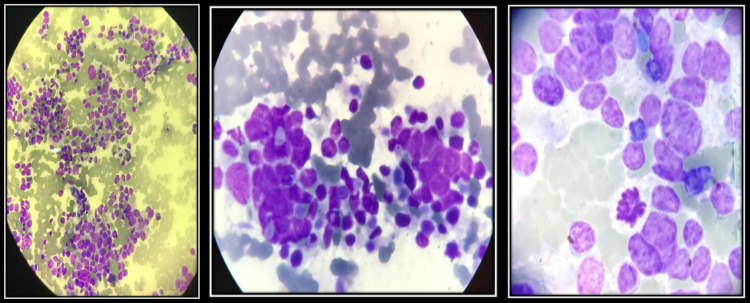
Microscopic features of small cell carcinoma lung showing clusters of small and round cells with scarce cytoplasm.

Histopathological examination of percutaneous biopsy from chest wall swelling and neck swelling confirmed the diagnosis as small cell (anaplastic or oat cell) lung carcinoma(SCLC) (Figure 3) with metastasis to ipsilateral cervical lymph nodes. After confirmation of diagnosis, metastatic workup was done with CT scan of abdomen and pelvis and MRI brain.

CT scan of abdomen and pelvis revealed a nodular mass with radiological features similar to a metastatic lesion in left adrenal gland but MRI brain was unremarkable. Patient received cisplatin and etoposide and was planned for subsequent palliative radiotherapy.

## Discussion

Chest wall swellings can arise from the skeletal or soft tissue structures of the thorax. Pulmonary and mediastinal neoplasms are common but seldom present initially as thoracic wall swelling [1]. The thoracic wall is considered to be involved when direct extension of a malignant pulmonary tumor is beyond the parietal pleura into any part of thoracic cage is microscopically confirmed [2]. Benign swellings like lipomas, chronic inflammatory granulomas and malignant swellings like sarcomas of chest wall, mediastinal and pleural neoplasms, and bronchogenic carcinoma invading chest wall should be kept in differential diagnosis. Chest wall swelling with underlying lung involvement is an uncommon presentation and initial presentation of small cell carcinoma lung as chest wall swelling is a very rare event and no such case was found with a similar presentation on PubMed database.

According to World Health Organization (WHO) 2004 classification scheme lung cancer is divided into two major histologic categories: non-small cell lung carcinoma (non-SCLC) and SCLC [3]. Only 13%-15% of all lung cancers are SCLC; however, SCLC are the most common primary pulmonary neuroendocrine neoplasm [4,5]. Cigarette smoking is the most common risk factor responsible for approximately 95% of cases, and SCLC has the strongest association with cigarette smoking [6].

Most of the patients initially present with complaints of cough, chest pain, hemoptysis, and dyspnea. Symptoms of systemic disease such as weight loss, fatigue, and anorexia are also seen at the time of diagnosis. Patients with invasive or advanced disease may present with specific symptoms such as dysphagia, hoarseness of voice and superior vena cava syndrome (in 10% cases) [7,8]. Numerous endocrine and neurological paraneoplastic disorders including Cushing syndrome, syndrome of inappropriate antidiuretic hormone secretion (SIADH), Lembert-Eaton syndrome, and limbic encephalitis have been described in association with SCLC [7]. No signs and symptoms suggestive of any of these paraneoplastic syndromes were found in our case. Metastatic disease is seen in approximately 60%-70% of patients at the time of diagnosis.

According to a modified version of the Veterans Administration Lung Cancer Study Group (VALSG) staging system SCLC is categorised as:

(A) Limited-stage SCLC (LS-SCLC),

(B) Extensive-stage SCLC (ES-SCLC).

However, the International Association for the Study of Lung Cancer (IASLC) has recommended that the American Joint Committee on Cancer (AJCC) tumor-node-metastasis (TNM) staging system for lung cancer to replace the modified VALSG system.

By definition, chest wall invasion is at least T3 disease. In this case (T3N3M1b), there was involvement of ipsilateral supraclavicular lymph nodes (N3) and a nodular lesion was seen in the left adrenal gland on CECT abdomen suggesting distant metastasis (M1b) in addition to chest wall invasion (T3).

Computed tomography (CT) is primarily used to evaluate the primary tumor and its intra-thoracic extent; however, PET/CT is more accurate in the staging of SCLC and can also be used to guide therapy and assess treatment response [9].

SCLC most commonly (in more than 95% cases) presents as a large mass centrally located within the lung parenchyma or as a mediastinal mass [10]. Its presentation as peripheral nodule (in less than 5% cases) is encountered less commonly [8]. Peripheral tumours present usually as a well-defined, homogeneous nodules or masses having speculations and lobular margins [11]. Surgical resection of SCLC is rarely performed and typically indicated in peripheral tumours. Therefore, peripherally located small cell lung carcinoma has better overall survival than central SCLC [12]. However, in our case, although the tumor was located in periphery unlike other peripheral small cell carcinoma it was not a well-circumscribed nodule and was invading chest well on initial presentation which might be due to a delay in diagnosis. This shows the importance of early diagnosis of small cell carcinoma which is a rapidly growing and very aggressive tumor. Chest wall invasion along with nodal and distant metastasis made the tumor unresectable in our case.

## Conclusions

This case highlights a rare presentation in which chest wall mass was the initial clinical presentation of peripheral small cell lung carcinoma. The overall prognosis of SCLC remains poor and five-year survival rate in LS-SCLC patients managed with curative-intent chemotherapy and radiation therapy is 10%-15%. Surgery has role only in early-stage peripherally located small cell lung carcinoma but most patients present with metastasis at the time of diagnosis. Clinical and basic research should continue to identify better treatment strategies that help to increase the duration of survival of patients with SCLC.
